# Characterization of oral candidiasis according to antiretroviral treatment status, immunological and virological profiles among HIV infected patients in two health facilities in Yaoundé-Cameroon: a cross-sectional and analytical study

**DOI:** 10.11604/pamj.2023.45.33.33714

**Published:** 2023-05-15

**Authors:** Joseph Fokam, Alex Durand Nka, Ezechiel Ngoufack Jagni Semengue, Cynthia Kelly Essono Asso’o, Jeremiah Efakika Gabisa, Aude Christelle Ka’e, Yagai Bouba, Willy Pabo, Buolikeze Kuoh Nji Geh, Davy Gouissi, Collins Ambe Chenwi, Michel Carlos Tommo Tchouaket, Aissatou Abba, Désiré Takou, Nadine Fainguem, Rachel Simo Kamgaing, Samuel Martin Sosso, Marie Elvire Nokam Abena, Alexis Ndjolo

**Affiliations:** 1 Chantal BIYA International Reference Centre for Research on HIV/AIDS Prevention and Management (CIRCB), Yaoundé, Cameroon,; 2 Faculty of Health Science, University of Buea, Buea, Cameroon,; 3 Ministry of Public Health, National HIV Drug Resistance Working Group, Yaoundé, Cameroon,; 4 Faculty of Medicine and Surgery, University of Rome “Tor Vergata”, Rome, Italy,; 5 Faculty of Sciences and Technology, Evangelical University of Cameroon, Mbouo-Bandjoun, Cameroon,; 6 Faculty of Medicine and Biomedical Sciences, University of Yaoundé I, Yaoundé, Cameroon,; 7 Faculty of Science, University of Buea, Buea, Cameroon,; 8 School of Health Sciences, Catholic University of Central Africa, Yaoundé, Cameroon

**Keywords:** HIV, oral candidiasis, viral load, CD4 count, Cameroon

## Abstract

**Introduction:**

oral candidiasis in HIV-disease generally indicates immune incompetence both among antiretroviral treatment (ART) naive and experienced patients. To optimize oral healthcare among people living with HIV (PLHIV) in sub-Saharan Africa (SSA), we sought to evaluate the type and distribution of oral candidiasis with respect to ART-profile and immuno-virological parameters among PLHIV in the Cameroonian context.

**Methods:**

a cross-sectional study was conducted among 163 patients (51 ART-naïve and 112 ART-experienced) residing in Yaoundé, Cameroon, from February through May 2019. Oral candidiasis was assessed, while viral load (VL) and CD4-count were measured on Abbott m2000rt and Cy-flow counter platforms, respectively. Data were analyzed using the Statistical Package for the Social Sciences (SPSS) v.21 with p<0.05 considered statistically significant.

**Results:**

in all, 18 cases of two forms of oral candidiasis were identified (13 erythematous and 5 pseudomembranous), with the majority, 27.7% (11/51), observed among ART-naïve patients against 6.3% (7/112) in ART-experienced (p=0.006). With respect to immuno-virological profile, 77.8% (14/18) and 22.2% (4/18) of cases were identified among participants with CD4<200 cells/mm^3^ and CD4>200 cells/mm^3^, respectively (p<0.0001). In the light of viral load, the occurrence of oral candidiasis was largely observed among subjects with VL≥1000 copies/ml, 83.3% (15/18), against 16.7% (3/18), with VL<1000 copies/ml, irrespective of the candidiasis form (p<0.0001).

**Conclusion:**

among PLHIV, erythematous and pseudomembranous candidiasis are commonly found in the absence of ART, driven by immunodeficiency and active viral replication. In spite of the protective role of ART, PLHIV experiencing immuno-virological failure should be referred for management of oral candidiasis.

## Introduction

Candidiasis is an opportunistic muco-cutaneous fungal infection caused by saprophytic fungi mainly *Candida albicans*. It represents more than 60% of yeasts isolated in humans [1,2]. Candida species are generally commensal but they become opportunistic pathogens mostly in people with a compromised immune system, such as persons on prolonged immune-suppressive therapy (65-88%), leukaemic patients undergoing radiotherapy (90%), and people living with HIV (PLHIV) (90-95%) [2]. Hence, candidiasis is an important comorbidity in HIV-infected patients worldwide [3]. Biological factors related to HIV involving CD4 count and viral load have an impact on oral mucosal variations [4]. Thus, the different forms of oral candidiasis pseudomembranous, acute atrophic (erythematous) candidiasis, etc., are likely to vary according to immuno-virological response [5-7]. Of note, HIV is responsible for nearly 37.7 million infections and 680 000 [480 000-1 million] deaths worldwide [8]. According to UNAIDS in 2020, 1.5 million [1.0 million-2.0 million] new HIV infections were recorded with 27.5 million people on ART [8], with an estimated 4.7 million [3.9 million-5.8 million] HIV infected people found within western and Central Africa. In Cameroon the prevalence of HIV is estimated at 2.7% [8]. Oral manifestations are among the earliest and most important indicators of HIV/AIDS infection [9]; thus, oral cavity pathologies can be a revelation of the disease. Among these oral manifestations, oral candidiasis is one of the most common comorbidity with HIV/AIDS [9]. The occurrence of opportunistic infections such as oral candidiasis is found in PLHIV with a CD4 count <200 cells/mm^3^ [10]. Apart from this CD4 value, oral candidiasis was also found in different forms in PLHIV. A study done in France in 2014, reported that pseudomembranous (25%) and erythematous (13.9%) candidiasis were the most encountered forms of oral candidiasis [11]. Meanwhile, in Cameroon, a study demonstrated the presence of periodontitis in HIV-infected people in Yaoundé, with a significantly high morbidity in those with a deleterious immune status (53.2%) but without exploration of the typology [12]. In order to limit HIV related oral disease and to optimize oral healthcare among PLHIV, we evaluated the type and distribution of oral candidiasis with respect to ART-profile including both immunological and virological profiles of PLHIV in two health facilities (Chantal Biya International Reference Centre for Research on HIV/AIDS Prevention and Management (CIRCB) and the Jamot Hospital of Yaoundé); which are respectively among the leading HIV/AIDS monitoring (viral load and CD4 count) and treatment sites in Yaoundé.

## Methods

**Study design and setting:** a cross-sectional and analytical study was conducted from February through May 2019 among PLHIV attending the Chantal BIYA International Reference Centre for Research on HIV/AIDS prevention and management (CIRCB), and the Jamot Hospital of Yaoundé. These two health facilities were chosen because of their strong involvement in the monitoring of people living with HIV in Cameroon and in the city of Yaoundé in particular.

**Sampling and enrollment criteria:** a statistical formula frequently used to determine sample size in medical studies [13]; together with the prevalence of HIV (2.7%) in Cameroon [8] were used to deduce our minimum sample size as follows:


$$n=\frac{{\left(Z\infty \right)}^{2}p\left(1-p\right)}{d^{2}}$$

where: Z_α_= standard normal variate (1.96 for a 95% confidence interval); p= prevalence of HIV in Cameroon (2.7); d= precision of the estimate (0.05). Substituting the figures in the above formula yields 40 (nearest whole number). Thus, a minimum of 40 participants was required. Participants enrolled were those who fulfilled the following criteria; a) be a person living with HIV/AIDS; b) understood the purpose of our finding and gave his/her consent to take part in the study; c) be at least 18 years old. We excluded patients who fell short of the aforementioned criteria. Also, those with other co-morbidities (diabetes mellitus, cancer), as well as those on prolong use of antibiotics (other than HIV- ART) and pregnant women were not included in this study.

**Clinical data collection and examination:** a structured-questionnaire was used to obtain sociodemographic characteristics of each participant. Treatment history (for those exposed to ART) and oral hygiene competence (brushing frequency) were equally assessed. We performed oral examination using a dental consultation tray, a tongue depressor, and a mouth mirror. Based on oral clinical presentation, location of lesions, presence or absence of bleeding, and whether the whitish coating had peeled off, the presence and type of oral candidiasis was determined. We assessed oral hygiene using Silness and Loe classification model [14]: where; 0= no plaque: good oral hygiene; 1) plaque detectable with a dental probe: average oral hygiene; 2) for visible plaque to the naked eye but not present in the interdental spaces; poor oral hygiene, and 3) visible plaque to the naked eye and present in the interdental spaces; poor oral hygiene.

**Assessment of immunological and virological parameters:** blood samples collected from HIV-infected patients were analyzed at the clinical diagnostic laboratory of CIRCB for CD4 cell count and plasma viral load. Briefly, CD4 count was performed using the Cyflow Counter-Sysmex Partec as per the manufacturer´s instructions [15]; viral load (VL) measurement was performed using the Abbott m2000rt Real Time PCR system as-per the manufacturer´s instructions [16], with a lower detection threshold of 40 copies/mL and an upper detection threshold of 10,000,000 copies/mL.

**Variables:** variables included gender, age, oral hygiene, treatment (compliance, ART exposure, ART regimen), and oral candidiasis. The rate and type of oral candidiasis was distributed according to gender, age, CD4 count, viral load, and treatment.

**Data processing and statistical analysis:** data were entered into Microsoft Excel 2013 and analyzed using the statistical software SPSS version 21 with results presented as mean, standard deviation, median, quartiles, frequencies and percentages. Bivariate analysis was done using Fischer´s exact and Chi-square test to determine oral candidiasis associated factors. Multivariate analysis was done using the logistic regression model. All p-values <0.05 were considered statistically significant.

## Results

**Characteristics of the study population:** a total of 163 participants were enrolled including 63 (38.7%) males and 100 (61.3%) females. Considering treatment-exposure, 51 (31.3%) were ART-naïve while 112 (68.7%) were ART-experienced patients. In the ART-naïve population, we had 20 (39.2%) males and 31 (60.8%) females, while in the ART-experienced population, 43 (38.4%) were males while 69 (61.6%) were females (Table 1). The mean age in both groups was 35.2±8.1 (ART-naive) and 42.1±9.5 years (ART-experienced). All ART-experienced patients enrolled for this study were under nucleoside reverse transcriptase inhibitors (NRTI) and non-nucleoside reverse transcriptase inhibitors (NNRTI) ART regimen. The most frequently used therapy registered was Tenofovir (TDF)+lamivudine (3TC)+Efavirenz (EFV), representing 70.5% (79/112) of ART experienced patients, while the least frequently used therapy was Zidovudine (AZT)+Lamivudine (3TC)+Nevirapine (NVP), 8.9% (10/112) (Table 1); 83.1% (93/112) of this sub-population was compliant to treatment. Overall, 88.0% (143/163) of PLHIV practiced oral hygiene averagely, while 8.0% (13/163) had poor oral hygiene practices; with merely 4.0% (7/163) of this study population having good oral hygiene practice (Table 1). Among PLHIV with poor oral hygiene, the plaque index according to Silness and Loe [12] was stage 3.

**Table 1:** characteristics of the study population

		Naive patients (N=51)	Patients on reverse transcriptase inhibitors (N=112)	Total (n%)	p-value
Gender n (%)	Female	31 (60.8)	69 (61.6)	100 (61.3)	p= 0.92
	Male	20 (39.2)	43 (38.4)	63 (38.7)	
Age (Years)	Median [IQR]	34 [28-39]	42 [36-49]	38 [32-44]	p<0.0001
	21-30	16 (31.4)	16 (37.5)	32 (19.6)	p<0.0001
	31-40	27 (52.9)	34 (30.4)	61 (37.4)	
	41-50	5 (9.8)	39 (34.8)	44 (26.9)	
	≥51	0 (0.0)	26 (23.2)	26 (15.9)	
ART Regimen	AZT + 3TC+ NVP	/	10 (8.9)	10 (8.9)	/
	TDF+3TC+EFV	/	79 (70.5)	79 (70.5)	
	TDF + 3TC + NVP	/	23 (20.5)	23 (20.5)	
HIV viral load (copies/ml)	Median [IQR]	211[ 40 - 16,653]	40 [ 40-838]	126 [40-8457]	p<0.0001
CD4 count (cells/mm^3^)	Median [IQR]	223 [119-442]	334 [168-528]	279 [144-485]	p= 0.02
Compliance to ART	YES	/	93 (83.0)	93 (83.0)	/
	NO	/	19 (17.0)	19 (17.0)	
Oral Hygiene	Good	2 (3.9)	5 (4.5)	7 (4.3)	p<0.0001
	Average	45 (96.1)	98 (87.5)	143 (88.3)	
	Poor	4 (0.0)	9 (8.0)	13 (8.7)	

/ is used here to mean no patient for this category

**Immuno-virological response:** the median CD4 count was 334[IQR: 168 - 528] cells/mm^3^ among ART-experienced patients against 223 [IQR: 119-442 cells/mm^3^ among ART naïve participants. meanwhile the median VL was 211 [IQR: 40 - 16 653] copies/mL among ART naïve patients compared to 40 [IQR: 40 - 838] copies/mL among ART experienced patients (Table 1).

**Candidiasis in the study population:** in all, the prevalence of oral candidiasis was 11.0% (18/163). Majority, 21.6% (11/51) was found among ART-naive PLHIV compared to the ART-experienced group that recorded merely 6.3% (7/112); p=0.006 (OR: 4.125: 95%CI: 1.49-11.39) (Table 2). Two forms of oral candidiasis were identified (erythematous and pseudomembranous forms). Erythematous candidiasis was predominant at 72.2% (13/18) and pseudomembranous candidiasis at 27.8% (5/18). Erythematous candidiasis was more predominant among ART-naïve, 13.7% (7/51), compare to 5.4% (6/112) among ART-experienced patients (p=0.11). Pseudomembranous candidiasis also prevailed among HIV-naïve, 7.8% (4/51) versus 2.7% (1/112) among ART-experienced patients at (p=0.03; OR=9.45; 95% CI: 1.028-86.783). In addition, we noticed non-compliant ART-experienced patients were more likely to developed oral candidiasis than their counterpart peers (21.0% vs. 3.2%) (p=0.028; OR=6.32; 95% CI: 1.30-30.56) (Table 2).

**Table 2:** oral candidiasis in the study population

		Presence of oral candidiasis (N=18)	Absence of oral candidiasis (N=145)	p-value
**Gender**	Female	9 (9.0)	91 (91.0)	0.29
	Male	9 (14.3)	54 (85.7)	
**Age (Years)**	21-30	4 (12.5)	28 (87.5)	0.76
	31-40	8 (13.1)	53 (86.9)	
	41-50	3 (6.8)	41(93.2)	
	≥51	3 (11.5)	23 (88.5)	
**Oral hygiene**	Good	0 (0.0)	6 (100.0)	0.24
	Average	15 (10.4)	129 (89.6)	
	Poor	3 (23.1)	10 (76.9)	
**CD4 count (cells/mm^3^)**	<200	14 (77.8)	46 (31.7)	p<0.0001
	≥200	4 (22.2)	99 (68.3)	
**HIV viral load (copies/ml)**	< 1000	3 (16.7)	109 (75.1)	p<0.0001
	≥ 1000	15 (83.3)	36 (24.8)	
**Antiretroviral treatment exposure**	ART naive	11 (21.6)	40 (78.4)	p=0.003
	Antiretroviral treatment treated	7 (6.3)	105 (93.7)	
**Compliance to antiretroviral treatment**	Yes	3 (3.2)	90 (96.8)	p=0.0034
	No	4(21.0)	15 (79.0)	
**Antiretroviral treatment regimen**	Zidovudine + Lamivudine+ Nevirapine	2 (20.0)	8 (80.0)	p= 0.1303
	Tenofovir (TDF) + lamivudine (3TC) + Efavirenz (EFV)	5 (6.3)	74 (93.7)	
	Zidovudine+Lamivudine+Nevirapine	0 (0.0)	23 (100.0)	

**Distribution of oral candidiasis according to CD4 count:** based on CD4 cell count, all oral candidiasis cases observed among naïve patients had CD4<200 cells/mm^3^, with 30.4% (7/23) and 17.4% (4/23) having the erythematous and pseudomembranous forms, respectively (Table 3). Among ART-experienced with erythematous candidiasis, 5.4% (2/37) and 5.3% (4/75) respectively had CD4<200 cells/mm^3^ and CD4>200 cells/mm^3^ (p=0.65). Those with the pseudomembranous form (CD4 counts 200 cells/mm^3^) represented 2.7% (1/37) and 0.0% for CD4>200 cells/mm^3^ (Table 3).

**Table 3:** distribution of the types of oral candidiasis according immuno-virological response among antiretroviral treatment naive and antiretroviral treatment treated patients

Naive patients	Erythematous candidiasis		P-value	Pseudomembranous candidiasis		p-value
CD4 count (cells/mm^3^)	Yes (n, %)	No (n, %)		YES (n, %)	NO (n, %)	
<200	7 (30.4)	16 (69.6)	0.002	4 (17.4)	19 (82.6)	0.03
≥ 200	0 (0.0)	28 (100)		0 (0.0)	28 (100)	
Viral load (copies/ml)						
< 1000	1 (3.8)	25 (96.1)	0.04	0 (0.0)	26 (100)	0.05
≥ 1000	6 (24.0)	19 (76.0)		4(16.0)	21(84.0)	
**Antiretroviral treatment treated patients**						
CD4 count (cells/mm^3^)						
<200	2 (5.4)	35 (94.5)	0.65	1 (2.7)	36 (97.3)	0.33
≥ 200	4 (5.3)	71 (94.6)		0 (00)	75 (100)	
**Viral load (copies/ml)**						
< 1000	2 (2.3)	84 (97.6)	0.02	0 (0.0)	86 (100)	0.23
≥ 1000	4 (15.4)	22 (84.6)		1 (3.8)	25 (96.1)	

**Distribution of oral candidiasis according to viral load:** among ART-naïve patients with VL<1000 copies/mL, 3.8% (1/26) were found to have erythematous candidiasis, whereas 24% (6/25) had VL≥1000 copies/mL (p=0.04; OR=7.89; 95%CI: 0.87-7.21) (Table 3). Pseudomembranous candidiasis among ART-naïve was found only in those with VL≥1000 copies/mL; (16%; 4/25) (p=0.05) (Table 3). Among ART-experienced patients, Pseudomembranous candidiasis was found in 3.8% (1/26) of those with a VL≥1000 copies/mL, while the erythematous form was present in 15.4% (4/26) of subjects with VL≥1000 copies/mL and 2.3% among those with viremia <1000 copies/mL (p=0.02) (Table 3).

**Distribution of oral candidiasis with respect to oral dental hygiene practice:** it was observed that all the diagnosed cases (18) of oral candidiasis were among individuals who practiced average, 10.4% (15/144) and poor, 23.1% (3/13), oral care, (p=0.24) (Table 2).

**Distribution of oral Candidiasis according to ART regimen:** according to ART regimen, patients on TDF+3TC+EFV had the highest number of oral candidiasis cases, 6.3% (5/79) followed by patients on AZT+3TC+NVP, 20.0% (2/10), p=0.11 (Table 2). Regarding the types of oral candidiasis, four cases of erythematous candidiasis were found among those on TDF+3TC+EFV and one case among patients on AZT+3TC+NVP. One case of pseudomembranous candidiasis was among patients on TDF+3TC+EFV regimen, p=0.21 (Table 2).

**Multivariate analysis:** after adjusting for gender, age, compliance (compliant and non-compliant), oral hygiene, CD4 count (<200 cells/mm^3^ and ≥200 cells/mm^3^), and viral load (<1000 copies/ml and ≥1000 copies/ml), we found that compliance (OR: 4.17; p=0.045) and viral load (OR: 0.003; p=0.007) were significantly associated with the occurrence of oral candidiasis.

## Discussion

This study was aimed at evaluating the relationship between oral candidiasis and HIV coinfection, taking into account ART-exposure, immunological and virological status of HIV-infected individuals. Women by virtue of biological difference (anatomy) and sociocultural reasons, are naturally more prone to STIs acquisition than their male counterpart [17,18]. Of the 163 PLHIV enrolled, more than 2/3 were ART experienced patients with a higher mean CD4 cell count than that observed among ART-naive participants, explained by the fact that HIV facilitates the selective loss of CD4 T-cells [19], while ART-initiation at an early stage helps avert this from occurring. This outcome is in line with a similar study conducted in Cameroon in 2019 [12], thus illustrating the positive impact of the “test and treat” strategy instituted since 2015 in the country is yielding and contributing to the fight against HIV/AIDS [20]. The overall prevalence of oral candidiasis was low (11.0%) compared to with previous studies [3,21-24]. This low prevalence could be explained by the difference in methods: while a Ghanaian study [23] employed deep mycological-findings, a Cameroonian study [25] focused solely on clinical manifestation to investigate oral candidiasis among PLHIV. The majority, 61.1% (11/18) of the 18 cases of oral candidiasis diagnosed were observed among ART-naive patients, highlighting the role of ART in preserving the immune system and thereby limiting the consequential evolution of opportunistic infections such as candidiasis [26,27]. Furthermore, two forms of oral candidiasis were identified in this study: erythematous and pseudomembranous, which are two key prognostic indicators in HIV infection and progression to AIDS [28,29]. Both forms of oral candidiasis were identified mainly among patients with severe immune suppression, which is an important predictor of opportunistic infections among PLHIV [30].

**Figure 1:**
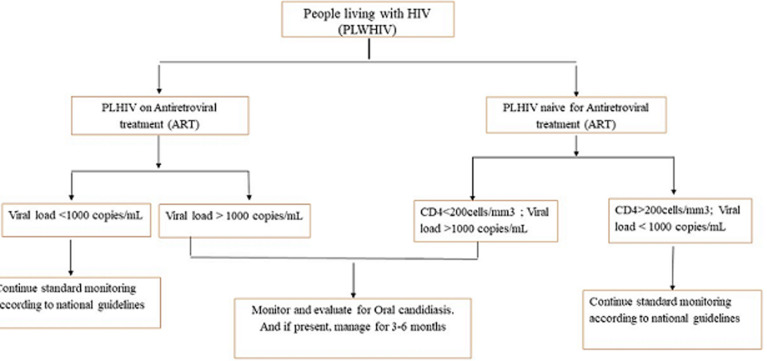
a proposed algorithm for the management of oral candidiasis among PLHIV in oral medicine

Actually, the relationship between candidiasis and CD4 cells is not direct. The major role in the resolution of oral candidiasis is mediated by a synergistic action between innate and acquired cell-mediated immunity [31]. Macrophages and T cells play an important role in proportions that depend on the site of the infection under consideration. Cell-mediated immunity involves natural killers, which appear to play a central role in anti-Candida immunity by delivering activating signals to immune cells via cytokine secretion [7,32]. In the presence of candidiasis, cytokines cause T cells to differentiate into Th1 cells under the action of IL-12 and TNF. The Th1 cells produced then initiates the mechanisms of phagocytosis of the genus Candida in the oral cavity through a polarized Thl-like protective response [33]. Independent studies carried out in India in 2011 and in France in 2014; showed an association between oral candidiasis and CD4 <200 cells/mm^3^ with OR= 6 and OR=3 respectively [11,34]. These findings are in line with the result we obtained in this study. Another study in South Africa showed that oral Candidiasis was negatively correlated with CD4 count in patients on treatment [35]; which proves that immunosuppression is a risk factor for the emergence of oral candidiasis. Regarding viremia, both forms of oral candidiasis found in this study were associated with high viremia among ART-naïve and ART-experienced patients. Our result is consistent with the study by Campo *et al*., who found that patients with high viremia had an eleven-fold increased risk of developing oral candidiasis compared to those virologically suppressed [36]. Considering our data, Figure 1 highlights a proposed algorithm for the monitoring and/or management of oral candidiasis among PLHIV. People on treatment with a viral load ≥1000 copies/ml should be monitored and/or managed for oral candidiasis for a period of 3 to 6 months. For treatment-naive patients with a viral load ≥1000 copies/ml and severe immune depression (CD4 <200 cells/mm^3^) should be managed and monitored for oral candidiasis.

**Limitations:** results obtained were based on two study sites and may not be applicable to the entire population/country. Also, no psychometric testing was used while collecting data and the ART-naïve population was small. There was lack of follow-up for these patients and the characterization of the oral microbiome in HIV-infected and HIV-uninfected subjects with their ART status was not done and could have been worth evaluating. Hence, studies covering these aspects should be carried out especially for the ART-naïve populations.

## Conclusion

In resource limited settings like Cameroon, oral candidiasis is still of concern among PLHIV with about 1/10 people affected. Erythematous and pseudomembranous candidiasis are commonly found in the absence of ART, driven by immunodeficiency and active viral replication as well as inadequate and poor oral hygiene. In spite of the protective role of ART, PLHIV experiencing immuno-virological failure should be referred for management of oral candidiasis.

### What is known about this topic

- *Oral candidiasis is an important indicator of suppressed immunity among people living with HIV;*
- *Antiretroviral treatment preserves the immune system and limit opportunistic oral candidiasis*.

### What this study adds

- *With the dispensation of the Cameroon´s “test and treat” strategy for HIV, oral candidiasis is now more associated with virological failure and not immunological failure;*
- *The occurrence of oral candidiasis among people living with HIV in the city of Yaoundé Cameroon is largely associated with poor and inadequate oral hygiene practice*.
